# Surgical treatment of inferior pole fractures of the patella: a systematic review

**DOI:** 10.1186/s40634-023-00622-y

**Published:** 2023-06-01

**Authors:** Chih-Hsun Chang, Chien-An Shih, Fa-Chuan Kuan, Chih-Kai Hong, Wei-Ren Su, Kai-Lan Hsu

**Affiliations:** 1 Department of Orthopaedic Surgery, National Cheng Kung University Hospital, College of Medicine, National Cheng Kung University, 138 Sheng-Li Rd, Tainan, Taiwan, R.O.C.; 2 Division of Orthopaedics, Department of Surgery, National Cheng Kung University Hospital Dou Liou Branch, National Cheng Kung University, Yunlin, Taiwan; 3 Skeleton Materials and Bio-Compatibility Core Lab, Research Center of Clinical Medicine, National Cheng Kung University Hospital, College of Medicine, National Cheng Kung University, Tainan, Taiwan; 4 Division of Traumatology, National Cheng Kung University Medical Center, Tainan, Taiwan; 5 Department of Orthopedic Surgery, Mayo Clinic, Rochester, MN USA

**Keywords:** Patellar fracture, Inferior pole, Lower pole, Distal pole, Systematic review

## Abstract

**Purpose:**

This study aimed to comprehensively review the existing evidence concerning surgical treatment of inferior pole fractures of the patella and to report the outcomes and complications of different fixation techniques.

**Method:**

This systematic review was conducted in accordance with the Preferred Reporting Items for Systematic Reviews and Meta-Analyses (PRISMA) guidelines. Searches of PubMed, Scopus, and Web of Science were conducted in March 2023. Studies were screened against predecided inclusion and exclusion criteria. The extracted data included fracture characteristics, surgical techniques, and radiographic and functional outcomes. The Methodological Index for Non-Randomized Studies (MINORS) quality assessment tool was used to assess the eligible literature. The primary outcome was postoperative range of motion of different surgical methods, and the secondary outcomes were other clinical results and complications.

**Results:**

A total of 42 studies satisfied all the inclusion criteria and were deemed suitable for review. Fourteen case–control studies and 28 case series were selected, for a total of 1382 patients with a mean age of 51.0 years (range = 11–90). The follow-up period ranged from 6 to 300 months. The surgical techniques were categorized based on the device used as follows: (1) rigid fixation device; (2) tensile fixation device; (3) mixed device; and (4) extra-patella device.

**Conclusion:**

Regarding the outcomes following surgical treatment of inferior pole fractures of the patella, the postoperative range of motion (ROM) of each technique ranged from 120° to 135°, with the exception of that involving the patellotibial wire which had poorer outcomes. The lowest functional score was also found in those using the patellotibial wire. Complications after surgery are rare, but approximately half of the patients required additional surgery for implant removal, particularly those whose initial surgery involved rigid fixation devices. It's worth noting that bony fragment excision is no longer recommended, and the combined use of multiple surgical devices is now more common.

**Supplementary Information:**

The online version contains supplementary material available at 10.1186/s40634-023-00622-y.

## Introduction

**Fig. 1:**
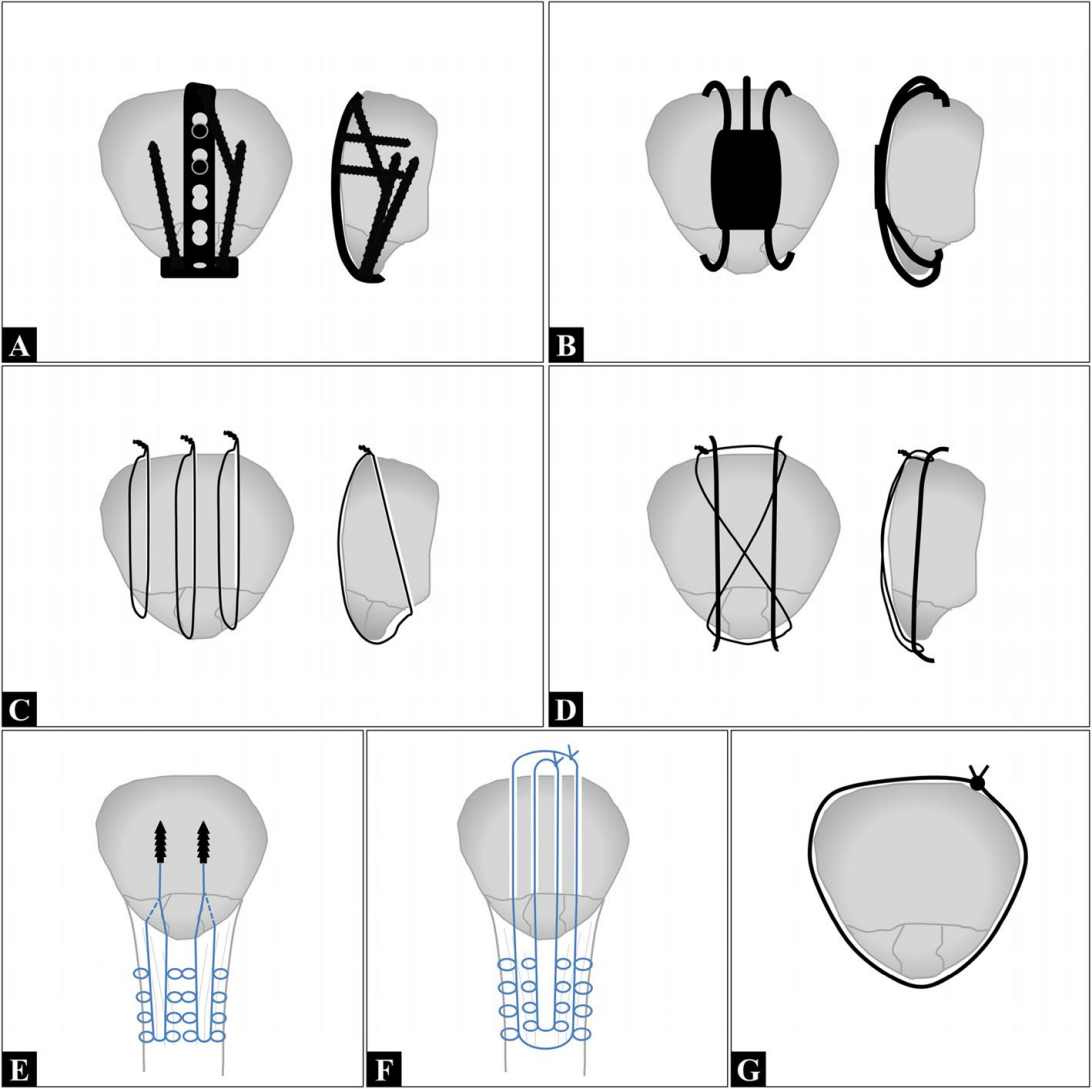
Surgical methods for treating inferior pole fractures of the patella: (**A**) plate and screw fixation; (**B**) concentrator fixation; (**C**) separated vertical wire (SVW); (**D**) tension band wire (TBW); (**E**) suture anchor (SA) fixation; (**F**) transosseous reattachment (TOR); (**G**) cerclage wire

Patellar fractures account for approximately 1% of all skeletal fractures in adults [11]. Inferior pole fracture of the patella, a type of patellar fracture in which the patella is extra-articularly avulsed by the patellar tendon, accounts for 5% to 22.4% of all patellar fractures [17]. Surgical treatment for displaced fractures of the inferior pole of the patella is recommended to restore the extensor mechanism of the lower extremity. However, a comminuted fracture complicates surgery. Experts have proposed various techniques for treating inferior pole fractures of the patella, including patella plates **(**Fig. 1A**)** or concentrators (Fig. 1B) [4, 6, 9, 18, 23, 25, 27, 30–32], and the use of separated vertical wiring (SVW; Fig. 1C**)** [5, 7, 10, 20, 33, 38, 42, 43, 45], tension band wiring (TBW; Fig. 1D**)** [2, 3, 6, 8, 16, 22, 26, 35, 41, 44, 46, 48], suture anchors (SA; Fig. 1E**)** [14, 17, 19, 26], and transosseous reattachment (TOR; Fig. 1F) with or without partial patellectomy [1, 2, 12, 14, 15, 17, 18, 21, 29, 31, 37, 47]. Additional techniques frequently used for augmentation include the use of cerclage wiring **(**Fig. 1G) [4, 10, 22, 23, 29, 38, 42, 44] and patellotibial wiring [21, 22, 37, 46]. Combinations of techniques are also employed to treat inferior pole fractures of the patella.

Although many case studies have indicated excellent outcomes of surgical methods, few studies have compared the clinical results of the aforementioned methods. The surgical methods in those case control studies varied. Furthermore, head-to-head comparisons are insufficient because of the various surgical methods employed. With a growing number of articles being published on this topic, a contemporary review of the literature is required to enable surgeons to select an appropriate surgical method and prevent possible complications. This paper provides a comprehensive review of the current evidence regarding the surgical treatment of inferior pole fractures of the patella. The aim of the study is to (1) investigate the postoperative range of motion (ROM) and (2) to report other functional outcomes and complications of different surgical technique.

## Method

The study protocol of this systematic review was registered on the International Prospective Register of Systematic Reviews (registration number: CRD42022363822).

### Search strategy

This systematic review was conducted in accordance with the Preferred Reporting Items for Systematic Reviews and Meta-Analyses guidelines [34]. This review searched PubMed, Scopus, and Web of Science using the following keywords in the title, abstract, and keywords sections of articles: “patella lower pole fracture,” “patella inferior pole fracture,” or “patella distal pole fracture.” The initial search was conducted in September 2022, and an updated search was conducted on March 7, 2023. After the database search, the keywords were then entered into Google Scholar to identify potentially relevant omitted studies. (Full search strategies were provided in the Appendix 1).

### Eligibility criteria

The selected studies satisfied the following criteria: (1) published in English, (2) included patients who underwent surgical fixation for inferior pole fractures of the patella, (3) classified as case–control studies and case series including 10 or more cases, and (4) reported validated outcome measures. This systematic review excluded (1) studies not published in English, (2) articles composed of abstracts only, conference abstracts, editorial comments, or expert opinion, (3) basic science studies, review articles, or technique notes, and (4) case reports that included less than 10 cases. Studies were assessed for eligibility in accordance with the criteria in Table 1.

**Table 1:** Inclusion and exclusion criteria used for study selection

Characteristics	Inclusion	Exclusion
Study availability	Full text available	Only abstract or title
Study type	Therapeutic	Basic research, systematic review, technique notes
Study content	Inferior pole fracture of the patella as main topic	Inferior pole fracture of the patella not the main subject
	Surgical treatment as main topic	Surgical treatment not the main subject
Case number	10 or more	Less than 10 cases
Follow-up	6 months or more	Less than 6 months
Language	English	Not English

The full texts were obtained and reviewed by two independent authors to assess eligibility. A senior author was consulted in cases of disagreement over study inclusion, and such disagreements were resolved by consensus. The references of the included studies were rescreened using the aforementioned method to prevent the omission of relevant articles.

### Quality assessment

This systematic review used the Methodological Index for Non-Randomized Studies (MINORS) quality assessment tool to assess the eligible literature, which assigns scores based on study design and level of bias. Comparative studies have a maximum score of 24, whereas noncomparative studies have a maximum score of 16. Two authors independently assessed the quality of each article.

### Data extraction

Data were extracted from the included studies by two authors independently in accordance with a predefined data extraction sheet. The recorded data included study design, sample size, patient demographic characteristics, fracture characteristics, surgical techniques, rehabilitation protocol, surgical time, time to union, postoperative radiological and functional outcomes, and complications. For postoperative rehabilitation, we defined ROM beginning sooner than 4 weeks postoperative as early ROM and that beginning after 4 weeks postoperative as late ROM. Similarly, partial weight-bearing (WB) beginning sooner than 2 weeks postoperative was defined as early WB, and that beginning after 2 weeks postoperative was defined as late WB. We did not record the timing of active ROM and full WB because some authors have indicated that these outcomes are dependent on healing status and thus differ by individual. Because of the various definitions of “complications” across studies, we defined major complications as follows: (1) a deep infection requiring surgical debridement or early removal of implants (ROIs), (2) loss of reduction necessitating revision osteosynthesis, and (3) other complications causing persistent functional impairment, such as neurovascular injury, recurrent giving way, and limping. Although ROIs after fracture healing were not defined as complications, researchers also extracted these data. The data were extracted separately for studies that used a different device in each treatment group.

### Statistical analysis

The primary outcome was postoperative ROM and the secondary outcomes were other clinical results of surgical methods, including operation time and functional score. For those outcomes, all continuous data were pooled, and a descriptive data analysis was implemented. The mean, standard deviation (SD), and range of the pooled outcome measures were determined. The SD was estimated from the range when not provided [40]. Studies that did not report the SD or the range were excluded from pooling. Pooled means and 95% confidence intervals were calculated for the outcome measures. For complications associated with surgical methods, the rates of complications and ROIs are listed. This review did not implement head-to-head comparisons between surgical methods because of the high heterogeneity in augmentation techniques and postoperative rehabilitation between studies.

Furthermore, for postoperative rehabilitation, we compared the timing of ROM exercise and WB using chi-squared tests. The results were obtained using SPSS (IBM, IL, US), with statistical significance indicated at *p* < 05.

## Results

**Fig. 2:**
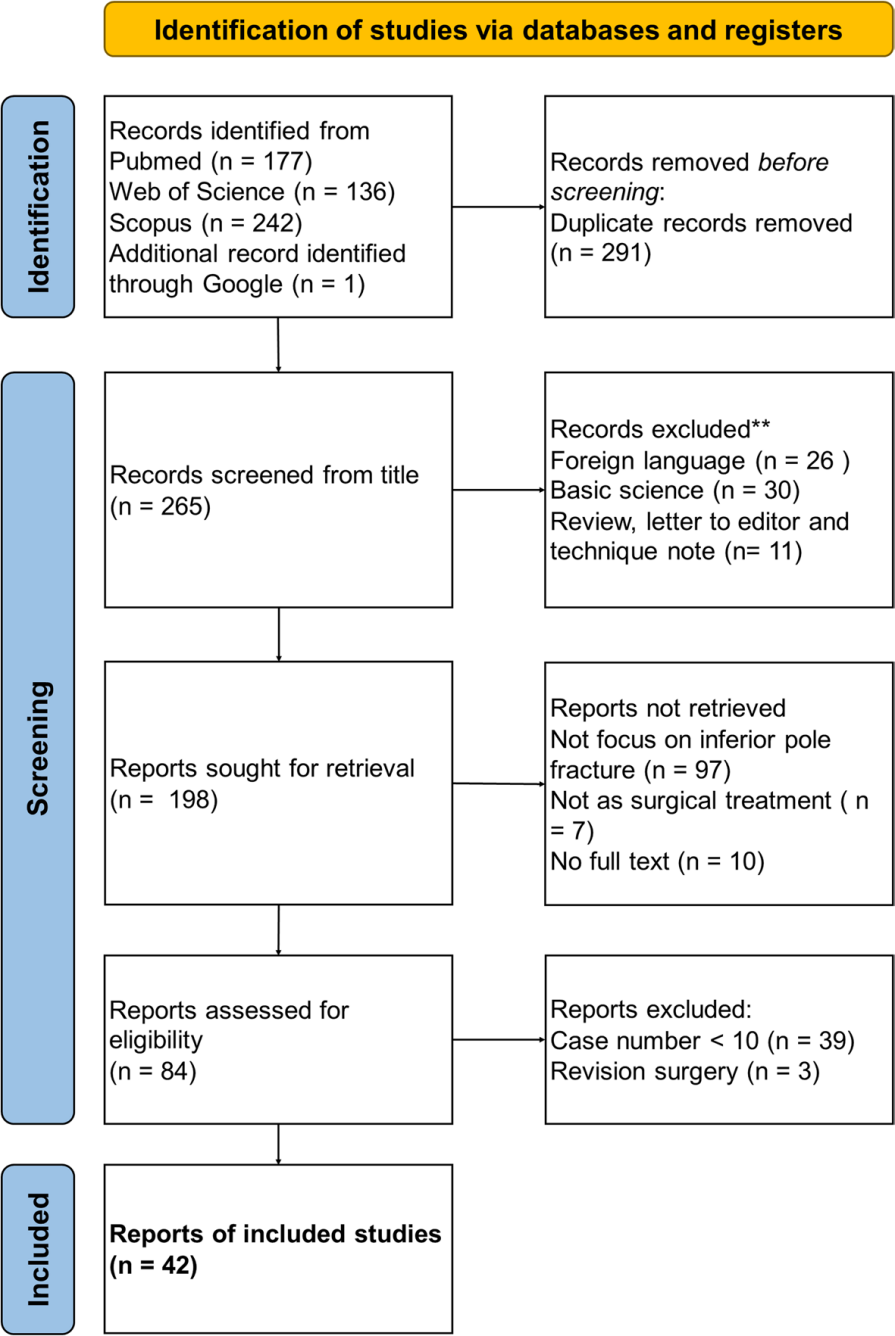
Flowchart of preferred reporting items for systematic reviews and meta-analyses guidelines

A total of 265 articles were obtained for review. According to our selection criteria, 42 studies were deemed suitable for inclusion **(**Fig. 2**)**. The detailed characteristics of the included studies are presented in Table 2. The number of publications increased between 2003 and 2023.

**Table 2:** General description of included studies

Author (Year)	Country	Evidence level	Method	Number of patients	Male	Female	Age (year)	Follow up (months)	MINORS score
Case control study									
Kastelec M 2004 [18]	Slovenia	RS	Plate	11	NM	NM	55.1 (11–77)	54 (29–87)	16/24
			TOR with partial patellectomy	13	NM	NM	50.5 (20–72)	56 (28–91)	
Matejcic A 2008 [31]	Croatia	RS	Plate	71	NM	NM	NM	NM	13/24
			TOR with partial patellectomy	49	NM	NM	NM	NM	
Kadar A 2016 [17]	Israel	RS	SA	27	15	12	52 ± 18	32.3 ± 11.5	17/24
			TOR	33	19	14	55 ± 20	41.6 ± 14.6	
Li J 2019 [22]	China	RS	TBW + cerclage wire + PT	30	18	12	52.6 ± 13.0	11.6 (9–15)	15/24
			TBW only	28	15	13	52.5 ± 11.6		
Zhang ZS 2020 [46]	China	RS	Screw TBW + PT	22	8	14	57.4 ± 13.7	15 ± 5.2	17/24
			Screw TBW	41	19	22	56.7 ± 13.8	18 ± 11.6	
Chang CH 2021 [2]	Taiwan	RS	TBW	30	10	20	59.7 ± 14.1	> 6	17/24
			TOR	25	13	12	55.3 ± 19.8	> 6	
Huang WZ 2021 [14]	China	RS	TOR	14	9	5	27.6 ± 10.6	22.6 ± 9.7	17/24
			SA	21	12	9	45.6 ± 11.5	18.7 ± 5.9	
Lu MK 2021 [26]	China	RS	TBW + SA	17	6	11	53.9 ± 11.1	24.2 ± 2.8	16/24
			TBW	20	10	10	54.4 ± 10.5	24.7 ± 2.5	
Yu H 2021 [45]	China	RS	SA	25	15	10	53.4 ± 8.6	18.2 ± 5.2	17/24
			SVM + cerclage wire	23	17	6	54.7 ± 8.1	16.7 ± 4.3	
Chen R 2022 [4]	China	RS	Concentrator	46	17	29	56.0 ± 10.0	> 12	17/24
			Concentrator + cerclage wire	48	19	29	55.8 ± 11.2	> 12	
Du B 2022 [6]	China	RS	TBW	21	NM	NM	51.4 ± 10.3	10.8 ± 1.1	17/24
			Plate + cerclage wire	28	NM	NM	54.8 ± 10.7	10.5 ± 1.2	
Kuo LY 2022 [21]	Taiwan	PS	TOR	15	3	12	58.7 ± 14.6	> 12	18/24
			TOR + PT	20	5	15	61.1 ± 15.5	> 12	
Xie J 2022 [41]	China	RS	SA + Fig. 8 wire	10	NM	NM	46.0 ± 19.5	7.9 ± 2.3	17/24
			TBW	18	NM	NM	47.6 ± 15.7	10.3 ± 4.2	
Park YG 2022 [35]	Korea	RS	TBW	35	20	10	35.4 ± 25.4	28.8 ± 3.6	17/24
			SA	28	19	9	34.9 ± 13.7	32.4 ± 8.4	
Case series									
Yang KH 2003 [43]	Korea	RS	SVM	25	19	6	36.8 ± 14.7	23.4 ± 14.1	7/16
Matejcic A 2006 [32]	Croatia	RS	Plate	51	38	13	46 (20–66)	60 (24–156)	7/16
Singh RP 2007 [37]	Nepal	RS	TOR + partial patellectomy + PT	14			35 (24–48)	36	6/16
Chang SM 2011 [3]	China	PS	Screw + TBW	10	6	4	59.8 ± 8.7	12	12/16
Kim YM 2011 [20]	Korea	RS	SVW	18	10	8	47.1 ± 13.3	29.9 ± 13.8	10/16
Liu XW 2011 [25]	China	RS	Concentrator	25	17	8	40.1 ± 18.5	26.0 ± 11.5	5/16
Song HK 2014 [38]	Korea	RS	SVM + cerclage wire	21	10	11	64.0 ± 11.7	25.4 ± 11.7	9/16
Matejcic A 2015 [30]	Croatia	RS	Plate	98	70	28	43.5 (18–69)	162 (12–300)	6/16
Oh HK 2015 [33]	Korea	RS	SVW + TOR	11	5	6	54.6 (23–74)	13 (10–23)	8/16
Fan J 2017 [7]	China	RS	Modified SVW	11	7	4	49.9 ± 18.2	17.2 (12–32)	7/16
Massoud EIE 2017 [29]	Egypt	PS	TOR + cerclage wire	23	5	18	83.4 ± 15.4	> 24	11/16
Yang X 2017 [44]	China	RS	TBW + cerclage wire	11	5	6	60.9 (29–81)	> 12	7/16
Cho JW 2018 [5]	Korea	RS	SVW + plate buttress	13	7	6	55.3 ± 12.36	13.5 ± 3.2	9/16
He S 2018 [10]	China	RS	SVW + cerclage wire	11	5	6	63.5 ± 11.0	21.7 (18–35)	10/16
Achudan S 2020 [1]	Singapore	RS	TOR + Fig. 8 wire	14	3	11	59.4 ± 11.7	6	6/16
Zhu W 2020 [48]	China	RS	TBW + buttress plate	17	8	9	52.8 ± 14.9	13.1 ± 1.4	8/16
Kim KS 2021 [19]	Korea	RS	SA	22	13	9	46 ± 20	25 ± 18	6/16
Yan SG 2021 [42]	China	RS	SVW + cerclage wire	20	10	10	54.0 ± 14.5	18.9 (12–36)	10/16
Jang JH 2021 [15]	Korea	RS	TOR + plate buttress	12	2	10	54.0	14.3	6/16
Hu JL 2022 [12]	China	PS	TOR + cerclage wire	22	13	9	47.8 ± 9.7	12 ± 4	10/16
Li M 2022 [23]	China	RS	Plate + cerclage wire	21	14	7	43.9 ± 7.8	12.6 ± 0.9	10/16
Pu SQ 2022 [36]	China	RS	Primary suture + ESF	11	6	5	39.0 ± 12.8	20.4 ± 7.6	10/16
Zhou M 2022 [47]	China	RS	TOR + suture bridge	18	10	8	50.1 ± 14.5	19.6 ± 5.0	10/16
Gao Z 2022 [8]	China	RS	TBW + TOR	15	8	7	43.4 ± 10.8	13.7 ± 1.7	9/16
Gu H 2022 [9]	China	RS	Plate + cerclage wire	16	8	8	55.6 ± 12.0	30.1 ± 5.3	10/16
Jian Z 2022 [16]	China	RS	TBW + cerclage wire	31	13	38	56.0 (32–78)	21.0 (18–35)	10/16
Liu CD 2023 [24]	China	RS	Screw + SA	22	8	14	55.0 (18–74)	16.7 ± 4.8	8/16
Ma XY 2023 [27]	China	RS	Plate	30	17	13	60.5 ± 10.5	13.8 ± 2.1	10/16

*ESF* external skeletal fixator, *NM* not mentioned, *PS* prospective, *PT* patellotibial wire, *RS* retrospective, *SA* suture anchor, *SVW* separated vertical wire, *TBW* tension band wire, *TOR* transosseous reattachment

### Qualitative synthesis

Fourteen case–control studies and 28 case series were selected. The included studies were composed of 38 retrospective studies, three prospective case series, and one prospective case–control study.

### Demographic data

This study included 1382 patients with a mean age of 51.0 (range = 11–90) years. The patients consisted of 606 men (53.07%), 536 women, and 240 patients with unreported sex. The follow-up period ranged from 6 to 300 months, with an average of 36.11 months.

### Fracture characteristics

Ten studies focused on comminuted fractures alone. Nine of the remaining 32 studies recorded comminuted fractures. The included studies recorded 107 (60.45%) comminuted fractures in 177 cases. Five studies measured the fracture gap, with the weighted average gap being 16.77 ± 11.75 mm. Four studies measured the absolute vertical length of the fragment, with a weighted average length of 14.99 ± 4.89 mm.

### Surgical method

The studies described a variety of surgical techniques, which can be divided into the following four major types (Appendix 2 and Table 3) based on the device used: (1) rigid fixation device, (2) tensile fixation device, (3) mixed device, and (4) augmentation with extra-patella device. Rigid fixation devices included those using plates and screws (*n* = 326, 8 studies; Fig. 1A) [6, 9, 18, 23, 27, 30–32] and concentrators (*n* = 119, 2 studies; Fig. 1B) [4, 25]. This group excluded devices using plates and screws as a buttress without penetration to the fracture site [5, 15]. Tensile fixation devices included those using SVW to repair the fracture and surrounding tendon (*n* = 153, 9 studies; Fig. 1C) [5, 7, 10, 20, 33, 38, 42, 43, 45], TOR with or without partial patellectomy (*n* = 238, 11 studies; Fig. 1D) [1, 2, 12, 14, 15, 17, 18, 21, 29, 31, 47], and SA (*n* = 133, 6 studies; Fig. 1E) [14, 17, 19, 35, 41, 45]. Mixed devices included those fixed using TBW (*n* = 294, 12 studies; Fig. 1F) [2, 3, 6, 8, 16, 22, 26, 35, 41, 44, 46, 48] or a similar technique with or without other augmentation (*n* = 22, 1 study) [24]. These devices combined rigid fixation devices (e.g., interosseous K-wire) and tensile fixation devices (e.g., figure-8 wire). Extra-patella devices included those fixing the patella to the tibia with a patellotibial wire (*n* = 86, 4 studies) [21, 22, 37, 46] or external skeletal fixators (ESF, *n* = 11, 1 study) [36]. Although cerclage wire **(**Fig. 1G) was used in the augmentation of 305 fractures, it has not been implemented as a standalone treatment device.

**Table 3:** Weighted averages and pooled data of surgical techniques

Technique	Number of patients	Surgery time (min)	ROM (degrees)	Functional score (Bostman score)	Major complications	Characteristics of complications	Removal of implants
Rigid fixation device	445	60.2	129.3	28.2	7/374	5 loss of reduction	**264/330**
		(*n* = 190)	(*n* = 95)	(*n* = 120)	(1.87%)	2 deep infections	**(80.00%)**
Tensile device dominant: SVM	153	66.6	131.5	28.9	2/153	1 loss of reduction	39/100
		(*n* = 92)	(*n* = 128)	(*n* = 153)	(1.31%)	1 deep infection	(39.00%)
Tensile device dominant: TOR	238	74.1	122.8	28.5	7/189	3 loss of reduction	23/125
		(*n* = 47)	(*n* = 128)	(*n* = 55)	(3.70%)	3 deep infections 1 paralysis	(18.40%)
Tensile device dominant: SA	133	53.1	124.0	28.1	**6/133**	1 loss of reduction	0/75
		(*n* = 73)	(*n* = 133)	(*n* = 56)	**(4.51%)**	5 deep infections	(0%)
Mixed device: TBW	294	70.4	120.4	27.5	9/294	6 loss of reduction	86/187
		(*n* = 137)	(*n* = 264)	(*n* = 153)	(3.06%)	3 deep infections	(45.99%)
Augmentation with PT wire	86	**80.3**	**112.2**	**25.8**	0/86		28/56
		**(*n* = 52)**	**(*n* = 72)**	**(*n* = 22)**	(0%)		(50.00%)

*PT* patellotibial wire, *ROM* range of motion, *SA* suture anchor, *SVW* separated vertical wire, *TBW* tension band wire, *TOR* transosseous attachment

### Postoperative rehabilitation

The timing of passive ROM exercise was recorded in 47 subgroups, with 36 (76.60%) having early ROM (Table 4). The timing of passive ROM exercise was significantly associated with the surgical method used (*p* = 0.008; Table 4). Surgeons who fixed fractures with TOR or SA tended to apply late ROM exercises to their patients. Furthermore, those who employed a single device to fix fractures also had a higher tendency to apply late ROM exercise compared with those who employed augmentation during fixation (*p* = 0.002). However, the final ROM was similar between patients with early ROM (124.39°) and those with late ROM (124.67°). The timing of WB was not associated with the surgical method used (*p* = 0.873) or with augmentation (*p* = 1.000).

**Table 4:** Rehabilitation protocol based on surgical method

Characteristics	Passive ROM exercise (*n* = 47)			Weight-bearing (*n* = 40)		
	Early (< 4 weeks)	Late (> 4 weeks)	*p* value	Early (< 2 weeks)	Late (> 2 weeks)	*p* value
Surgical technique			.008			.873
Rigid fixation device	9	0		5	1	
Tensile device dominant: SVM	6	1		4	2	
Tensile device dominant: TOR	5	5		7	2	
Tensile device dominant: SA	2	4		3	1	
Mixed device	10	1		9	2	
Augmentation with PT wire	4	0		4	0	
Augmentation or not			.002			1.000
Single device	14	10		16	4	
Additional augmentation	22	1		16	4	

*PT* patellotibial wire, *ROM* range of motion, *SA* suture anchor, *SVW* separated vertical wire, *TBW* tension band wire, *TOR* transosseous attachment

### Radiographic outcomes

A total of 29 studies assessed the radiographic union, and 19 reported an accurate union time. The weighted average union time was 10.44 ± 3.30 weeks. Although 13 studies investigated postoperative patella heights, the measurement methods varied. The methods used included the Insall–Salvati ratio (6 studies), the Blackburne–Peel ratio (3 studies), the Caton–Deschamps ratio (1 study), the plateau–patella angle method (1 study), and measurement of patella height only (2 studies).

### Postoperative ROM

Although 38 studies measured the ROM, an accurate degree measurement was recorded in only 32 studies with 42 subgroups. The weighted average ROM of different methods is presented in Table 3. The lowest ROM (ROM = 112.2°) was recorded in patients who underwent augmentation with a patellotibial wire. We also compared the postoperative ROM between those who underwent early ROM exercise (124.39°) and late ROM exercise (124.67°). However, the results indicated no significant difference between the two groups.

### Functional outcomes

Thirty-eight studies measured functional scores, with 29 reporting a range or SD. The functional scores used in the studies included the Bostman score (27 studies), the Lysholm score (5 studies), the patellofemoral score (3 studies), the modified Cincinnati Knee Rating System (3 studies), the Kujala score (1 study), the 12-Item Short Form Survey (1 study), the International Knee Documentation Committee Subjective Knee Form (1 study), and the Knee Injury and Osteoarthritis Outcome Score (1 study). The weighted average functional score of different methods is listed in Table 3. The lowest Bostman score was noted in patients who underwent augmentation with a patellotibial wire.

### Complications

All studies except one reported complications. Major complications were reported in 31 out of 1,262 cases (2.46%). The most common complications included loss of reduction requiring revision osteosynthesis (*n* = 16, 1.27%) and deep infection requiring surgical debridement (*n* = 14, 1.11%). A high complication rate was recorded in SA groups (4.5%).

### ROIs

Nine case–control studies and 18 case series reported ROI rates; in this study cohort, 451 of 884 (51.01%) patients underwent surgery to remove implants. The reasons for ROIs included patient request (*n* = 43, 8.70%), implant-related irritation (*n* = 36, 7.29%), implant breakage (*n* = 33, 6.68%), staged surgery (*n* = 11, 2.23%), and inferior patella after patellotibial wiring (*n* = 6, 1.21%). The reasons for ROIs in the remaining 321 patients were not recorded. The rates of ROIs in each subgroup are presented in Table 3. The highest ROI rate was noted in the rigid fixation device group (80.00%).

## Discussion

The present systematic review revealed that studies have published a wide range of surgical techniques for the treatment of inferior pole fractures of the patella. This comprehensive review reveals good and excellent outcomes following most surgical methods. In addition, the results demonstrate the drawbacks of certain surgical methods and identify those with a high ROI rate.

This study revealed two primary findings with respect to surgical methods. First, although partial patellectomy has been used in clinical practice, its application in recent decades in rare. Removal of fragments and shortening of the patella length result in increased patellofemoral pressure [28] and poor functional outcomes [31]. The goals of current surgical techniques are to not only restore the extensor mechanism and achieve solid bony union but to also reduce complications. Second, the combination of multiple surgical devices is common. Cerclage wire is the device most commonly employed for augmentation, even after rigid fixation with a plate or concentrator. Other devices include a patellotibial wire, buttress plate without screw, and figure-8 suture. The use of a cerclage wire may be attributable to the high comminution rate (60.45%) and high loading after fixation, which may force the surgeon to use maximum strength to achieve adequate fixation.

Another valuable finding is that postoperative rehabilitation significantly depended on the surgical method, particularly the timing of passive ROM. Surgeons who employed TOR or SA tended to apply late ROM exercise in patients. Those techniques may contribute to concern of loss of reduction because they involve employing relatively weak nonmetal devices. Thus, surgeons should employ more rigid fixation devices or use augmentation techniques to avoid prolonged immobilization and encourage patients to start ROM earlier.

The average union time in the pooled data was 10.44 ± 3.30 weeks, which is similar to that for transverse fracture of the patella [13, 39]. However, 29 studies assessed radiographic union, and only 19 reported an accurate union time. One possible explanation is the difficulty in assessing the healing status with many implants around the fracture site. However, nonunion does not always contribute to functional loss. Kadar et al. and Achudan et al. have reported cases of nonunion without loss of the extension mechanism [17]. Chang et al. also demonstrated the vital role of fibrous union in postoperative stability and explained why no obvious function loss was noted in cases with fracture nonunion [2]. Liu et al. assessed not only radiological bony union but also clinical bony union [24]. Therefore, the present study defined the loss of reduction as a complication only when it was revised or resulted in persistent functional loss.

The Bostman score is most widely used for the assessment of functional outcomes following surgical fixation of the inferior pole fractures of the patella. The pooled data indicate good to excellent results for different surgical techniques with the exception of those that employed a patellotibial wire. The Lysholm score and patellofemoral score also indicated good to excellent results. The average ROM resulting from different surgical techniques ranged from 120° to 135°. However, the average postoperative ROM measured was only 112.2° for fractures fixed with patellotibial wire. This reduced functional score and ROM may result from prolonged restriction in flexion. Treatment providers should consider aggressive rehabilitation or the removal of patellotibial wires in patients with protracted knee stiffness.

The surgical treatment of inferior pole fractures of the patella has a low postoperative complication rate (2.46%). A loss of reduction occurred most often, which may be related to the high rate of comminution and high load on the patellar tendon. Because of the risk of losing reduction, surgeons may employ additional augmentation or postpone the timing of ROM training. In addition, Chang et al. found that a preoperative fracture gap larger than 30 mm significantly increased the postoperative loss reduction rate [48]. That study’s results indicated that surgeons must modify their rehabilitation protocol not only based on the surgical method but also the fracture severity.

Furthermore, 50.91% of patients received ROIs after bony union. The subgroup analysis identified the highest rate of ROIs in the rigid fixation group (80.00%). The bulkiness of the implants and their superficial placement may explain this finding. However, advances in technology leading to the development of low-profile devices may help decrease the irritation caused by implants. Ma et al. and Du et al., who employed low-profile plates to treat inferior pole fractures of the patella, indicated that ROIs were rarely required [7, 10].

The results of this systematic review are encouraging for surgeons because the postoperative functional outcomes ranged from good to excellent and complications were rare. Surgeons can select the appropriate surgical method based on their experience and the availability of implants. However, postoperative rehabilitation, particularly the timing of ROM, should be adjusted in accordance with surgical technique and fracture severity. Surgeons who use a patellotibial wire should be aware of the possible outcomes of inferior ROM and functional score. Finally, patients must be informed of the high ROI rate prior to surgery.

This systematic review is the first to classify and analyze the different surgical methods for inferior pole fractures of the patella. However, this review has several limitations. The lack of randomized controlled trials did not allow for a meta-analysis. Therefore, this review is unable to assert a definitive conclusion on different surgical techniques. We conducted a qualitative systematic review with pooled descriptive data with respect to each study’s published techniques. In addition, the qualitative synthesis indicated a predominance of studies with evidence levels of V and IV and a heterogenous MINORS score. The risk of bias indicated by the MINORS scores may cast doubt on the impartiality of the published techniques. Considerably heterogenous data on ROIs were noted, particularly regarding the reasons for and complications of this procedure in different techniques. Furthermore, selected studies failed to thoroughly describe the consequences of ROIs or how to avoid them.

## Conclusion

Regarding the outcomes following surgical treatment of inferior pole fractures of the patella, the postoperative ROM of each technique ranged from 120° to 135°, with the exception of that involving the patellotibial wire. The lowest functional score was also found in those using the patellotibial wire. Complications after surgery are rare, but approximately half of the patients required additional surgery for implant removal, particularly those whose initial surgery involved rigid fixation devices. Besides, excision of bony fragments is no longer recommended, and the combined use of multiple surgical devices is common.

## Supplementary Information

**Additional file 1:** **Appendix 1.** Search strategy and results.

**Additional file 2:** **Appendix 2.** Detail of the articles.
