# Supplementary material for: Surgical treatment of inferior pole fractures of the patella: a systematic review
Source: J Exp Orthop. 2023 Jun 1;10:58. doi: 10.1186/s40634-023-00622-y (PMC10234962; doi:10.1186/s40634-023-00622-y)
Supplement: Supplementary file 1 — Additional file 1: Appendix 1. Search strategy and results. [file 40634_2023_622_MOESM1_ESM.docx]

**Search in Pubmed**

| Strategy | ((patella lower pole fracture) OR (patella inferior pole fracture)) OR (patella distal pole fracture) in all field |
| --- | --- |
| Result  (n=177) | {Yan, 2023 #13439;Vesseur, 2023 #13399;Shimasaki, 2023 #13397;Mei, 2023 #13415;Ma, 2023 #13407;Liu, 2023 #13398;Liu, 2023 #13402;Englert, 2023 #13403;Chun, 2023 #13396;Alley, 2023 #13400;Zhu, 2022 #13413;Zhu, 2022 #13423;Zhou, 2022 #13418;Xie, 2022 #13430;Tengler, 2022 #13443;Seggewiss, 2022 #13405;Raja, 2022 #13434;Pu, 2022 #13414;Park, 2022 #13404;Negrao, 2022 #13406;Lowe, 2022 #13417;Lin, 2022 #13428;Li, 2022 #13409;Li, 2022 #13420;Kuo, 2022 #13419;Kulkarni, 2022 #13411;Jian, 2022 #13401;Hu, 2022 #13424;Hannah, 2022 #13426;Gu, 2022 #13410;Gao, 2022 #13412;Gao, 2022 #13416;Du, 2022 #13408;Devana, 2022 #13427;Chen, 2022 #13425;Berninger, 2022 #13422;Yu, 2021 #13429;Xie, 2021 #13445;Wang, 2021 #13432;Tomar, 2021 #13441;Tanpowpong, 2021 #13449;Sousa, 2021 #13469;Sim, 2021 #13457;Shi, 2021 #13435;O'Donnell, 2021 #13437;Meng, 2021 #13460;Lu, 2021 #13438;Kim, 2021 #13454;Kfuri, 2021 #13450;Jang, 2021 #13444;Howatt, 2021 #13436;He, 2021 #13446;Harna, 2021 #13433;Fehske, 2021 #13451;Deng, 2021 #13440;Chang, 2021 #13442;Carter, 2021 #13421;Bulaid, 2021 #13447;Bae, 2021 #13431;Zhu, 2020 #13465;Zhang, 2020 #13466;Yu, 2020 #13462;Wilding, 2020 #13453;Sun, 2020 #13458;Siddiqui, 2020 #13456;Schmidt-Hebbel, 2020 #13461;Misir, 2020 #13467;Mehta, 2020 #13463;Gupta, 2020 #13455;Guler, 2020 #13459;Fan, 2020 #13464;Callahan, 2020 #13448;Achudan, 2020 #13452;Shea, 2019 #13470;Schutte, 2019 #13471;Li, 2019 #13478;Jang, 2019 #13468;Siljander, 2018 #13477;Paxinos, 2018 #13480;Meng, 2018 #13475;He, 2018 #13476;Gu, 2018 #13473;Cusano, 2018 #13482;Cho, 2018 #13481;Cereijo, 2018 #13472;Alassaf, 2018 #13474;Zhang, 2017 #13492;Yang, 2017 #13483;Wight, 2017 #13479;Swensen, 2017 #13484;Siljander, 2017 #13485;Massoud, 2017 #13487;Damrow, 2017 #13488;Zhang, 2016 #13491;Yoon, 2016 #13493;Wild, 2016 #13490;Verbeek, 2016 #13489;Tsubosaka, 2016 #13486;Kataoka, 2016 #13494;Kadar, 2016 #13501;Hermansen, 2016 #13498;Aksahin, 2016 #13504;Sun, 2015 #13502;Sampath, 2015 #13496;Potini, 2015 #13500;Pascarella, 2015 #13503;Oh, 2015 #13497;Matejcic, 2015 #13495;Lorich, 2015 #13499;Song, 2014 #13506;Ro, 2014 #13514;Matsuo, 2014 #13507;Kimball, 2014 #13508;Egol, 2014 #13505;Yoshioka, 2013 #13509;Vidovic, 2013 #13513;Tang, 2013 #13512;Schuttrumpf, 2013 #13516;Maniar, 2013 #13510;Lazaro, 2013 #13519;Lazaro, 2013 #13511;Kang, 2013 #13515;Harris, 2013 #13528;Bazylewicz, 2013 #13517;Peek, 2012 #13526;Neill, 2012 #13520;Maniar, 2012 #13522;Huang, 2012 #13518;Hassan, 2012 #13525;Beamish, 2012 #13523;Allen, 2012 #13521;Stocker, 2011 #13533;Kim, 2011 #13524;Chang, 2011 #13529;Bright, 2011 #13530;Barlow, 2011 #13527;Zhang, 2010 #13531;Zhang, 2010 #13534;Anand, 2010 #13532;Iwamoto, 2009 #13536;Dietz, 2009 #13535;Matejcic, 2008 #13538;Langley-Hobbs, 2008 #13537;Uvaraj, 2007 #13540;Singh, 2007 #13539;Khanna, 2007 #13541;Matejcic, 2006 #13542;Veselko, 2005 #13545;Seybold, 2005 #13544;Kumar, 2005 #13547;Fazal, 2005 #13546;Yeung, 2004 #13549;Rasit, 2004 #13543;Kastelec, 2004 #13548;Yang, 2003 #13550;Hansen, 2003 #13551;Ziring, 2002 #13552;Atkinson, 2001 #13553;Lebel, 1999 #13554;Ishaque, 1999 #13555;Harwin, 1998 #13556;Benson, 1998 #13557;Arredondo, 1997 #13558;Veselko, 1996 #13559;Shands, 1995 #13560;Berg, 1995 #13561;Kolndorfer, 1994 #13564;Derwin, 1994 #13563;Bates, 1994 #13562;Rink, 1991 #13566;Dubrov, 1991 #13565;Rockett, 1990 #13567;Hozack, 1988 #13568;Goldberg, 1988 #13569;Heckman, 1984 #13570;Ogden, 1982 #13571;Sugiura, 1972 #13572} |

**Search in Web of Science**

| Strategy | ((patella lower pole fracture) OR (patellar inferior pole fracture)) OR (patella distal pole fracture) in Title/Keywords/Abstract |
| --- | --- |
| Result  (n=136) | {Achudan, 2020 #14536;Aksahin, 2016 #14564;Alassaf, 2018 #14549;Allen, 2012 #14590;Anand, 2010 #14598;Atkinson, 2001 #14617;Bae, 2021 #14516;Bates, 1994 #14624;Bazylewicz, 2013 #14580;Beltran, 2012 #14586;Benson, 1998 #14620;Berg, 1995 #14623;Berninger, 2022 #14511;Bradko, 2018 #14552;Bright, 2011 #14595;Bui, 2018 #14548;Bulaid, 2021 #14529;Callahan, 2020 #14530;Capps, 1994 #14625;Chang, 2021 #14523;Chang, 2011 #14594;Cho, 2018 #14555;Cho, 2011 #14591;Cusano, 2018 #14556;Damrow, 2017 #14560;Deng, 2021 #14522;Derwin, 1994 #14626;Devana, 2022 #14513;Dietz, 2009 #14600;Drabicki, 2006 #14608;Du, 2022 #14500;Dubrov, 1991 #14627;Dy, 2012 #14588;Fan, 2017 #14563;Fan, 2020 #14539;Fazal, 2005 #14611;Fehske, 2021 #14531;Gao, 2022 #14501;Hambright, 2017 #14561;Hannah, 2022 #14509;Hansen, 2003 #14615;Harna, 2021 #14518;Harris, 2013 #14576;Harwin, 1998 #14619;He, 2021 #14526;He, 2018 #14553;Hempfling, 2018 #14554;Hermansen, 2016 #14568;Hu, 2022 #14512;Huang, 2012 #14585;Jang, 2021 #14524;Jang, 2019 #14542;Jian, 2023 #14494;Kadar, 2016 #14569;Kan, 2015 #14573;Kang, 2013 #14581;Kastelec, 2004 #14612;Kfuri, 2021 #14525;Khanna, 2007 #14604;Kim, 2021 #14533;Krkovic, 2007 #14605;Kumar, 2005 #14610;Kuo, 2022 #14508;Langley-Hobbs, 2008 #14602;Li, 2019 #14543;Li, 2022 #14510;Li, 2022 #14502;Liu, 2011 #14593;Lowe, 2022 #14505;Lu, 2021 #14519;Ma, 2023 #14499;Maniar, 2013 #14578;Maniar, 2012 #14589;Massoud, 2017 #14559;Matejcic, 2015 #14572;Matejcic, 2008 #14601;Matejcic, 2006 #14607;McElvany, 2013 #14577;Mei, 2023 #14496;Memisoglu, 2011 #14592;Misir, 2020 #14538;Negrao, 2022 #14498;O'Donnell, 2021 #14520;O'Sullivan, 2010 #14599;Oh, 2015 #14570;Paxinos, 2018 #14550;Peek, 2012 #14587;Petermann, 2001 #14618;Potini, 2015 #14571;Pu, 2022 #14504;Raja, 2022 #14517;Ro, 2014 #14575;Sarierler, 2013 #14583;Schmal, 2010 #14597;Schutte, 2019 #14545;Schutte, 2019 #14546;Schuttrumpf, 2013 #14579;Seggewiss, 2022 #14497;Senaran, 2007 #14606;Sessions, 2018 #14551;Seybold, 2005 #14609;Shands, 1995 #14622;Shea, 2019 #14544;Shimasaki, 2023 #14495;Siljander, 2018 #14547;Siljander, 2017 #14558;Sim, 2021 #14537;Song, 2014 #14574;Sousa, 2021 #14527;Stocker, 2011 #14596;Sun, 2020 #14534;Swensen, 2017 #14557;Tanpowpong, 2021 #14528;Tibone, 1981 #14628;Uvaraj, 2007 #14603;Verbeek, 2016 #14565;Veselko, 1996 #14621;Vesseur, 2023 #14493;Vidovic, 2013 #14582;Visser, 2013 #14584;Wild, 2016 #14566;Wilding, 2020 #14535;Wong, 2020 #14532;Xie, 2022 #14515;Yan, 2023 #14521;Yang, 2003 #14614;Yeung, 2004 #14613;Yoon, 2016 #14567;Yu, 2021 #14514;Yu, 2020 #14540;Zhang, 2017 #14562;Zhang, 2020 #14541;Zhou, 2022 #14506;Zhu, 2022 #14507;Zhu, 2022 #14503;Ziring, 2002 #14616} |

**Search in Scopus**

| Strategy | ((patella lower pole fracture) OR (patellar inferior pole fracture)) OR (patella distal pole fracture) in Title/Keywords/Abstract |
| --- | --- |
| Result  (n=242) | {Kaye, 1971 #14870;Sugiura, 1972 #14869;Labitzke, 1973 #14868;Marti, 1973 #14867;Rosenthal, 1977 #14866;Tibone, 1981 #14865;Dickason, 1982 #14864;Ogden, 1983 #14863;Baldini, 1984 #14862;Heckman, 1984 #14861;Liang, 1987 #14860;Goldberg, 1988 #14858;Hozack, 1988 #14859;Renstrom, 1989 #14857;Gardiner, 1990 #14856;Maffulli, 1990 #14855;Rockett, 1990 #14854;Bates, 1994 #14853;Capps, 1994 #14849;Derwin, 1994 #14851;Kölndorfer, 1994 #14850;Tani, 1994 #14852;Berg, 1995 #14847;Satku, 1995 #14846;Shands, 1995 #14848;Veselko, 1996 #14845;Arredondo, 1997 #14843;Ferrari, 1997 #14844;Benson, 1998 #14841;Harwin, 1998 #14842;Powell, 1998 #14840;Ishaque, 1999 #14838;Ishaque, 1999 #14834;Ishaque, 1999 #14837;Kondo, 1999 #14836;Lebel, 1999 #14839;Nakagawa, 1999 #14835;Ishaque, 2000 #14833;McWilliams, 2000 #14832;Schmal, 2000 #14830;Ziring, 2000 #14831;Atkinson, 2001 #14827;Liu, 2001 #14829;Petermann, 2001 #14828;Harty, 2002 #14825;Mauch, 2002 #14826;Ziring, 2002 #14824;Hansen, 2003 #14821;Hansen, 2003 #14823;Yang, 2003 #14822;Kastelec, 2004 #14820;Rasit, 2004 #14819;Singh, 2004 #14818;Yeung, 2004 #14817;Fazal, 2005 #14815;Kumar, 2005 #14814;Polykandriotis, 2005 #14813;Seybold, 2005 #14812;Veselko, 2005 #14816;Klerx-Melis, 2006 #14810;Matejčić, 2006 #14811;Capiola, 2007 #14808;Khanna, 2007 #14804;Krkovic, 2007 #14805;Lai, 2007 #14807;Senaran, 2007 #14809;Singh, 2007 #14806;Uvaraj, 2007 #14803;Langley-Hobbs, 2008 #14800;Matejčič A, 2008 #14802;Mayayo Sinués, 2008 #14801;Brooks, 2009 #14797;Dietz, 2009 #14798;Iwamoto, 2009 #14799;Anand, 2010 #14793;Cakici, 2010 #14794;Havlas, 2010 #14791;Schmal, 2010 #14792;Zhang, 2010 #14796;Zhang, 2010 #14795;Barlow, 2011 #14784;Bright, 2011 #14788;Chang, 2011 #14787;Chen, 2011 #14783;Cho, 2011 #14785;Liu, 2011 #14786;Memisoglu, 2011 #14789;Stocker, 2011 #14790;Allen, 2012 #14782;Beamish, 2012 #14779;Beltran, 2012 #14775;Hassan, 2012 #14780;Huang, 2012 #14776;Maniar, 2012 #14781;Neill, 2012 #14778;Peek, 2012 #14777;Bazylewicz, 2013 #14765;Gao, 2013 #14771;Harris, 2013 #14759;Jones, 2013 #14764;Kamath, 2013 #14760;Kang, 2013 #14768;Lazaro, 2013 #14762;Lazaro, 2013 #14766;Mak, 2013 #14767;Maniar, 2013 #14761;McElvany, 2013 #14773;Sarierler, 2013 #14770;Schüttrumpf, 2013 #14763;Tang, 2013 #14774;Vidović, 2013 #14772;Voon, 2013 #14769;Alagöz, 2014 #14753;Egol, 2014 #14750;Kimball, 2014 #14755;Matsuo, 2014 #14757;O'Brien, 2014 #14749;Pishnamaz, 2014 #14752;Pishnamaz, 2014 #14751;Ro, 2014 #14758;Song, 2014 #14756;Song, 2014 #14754;Hankemeier, 2015 #14748;Lorich, 2015 #14742;Lorich, 2015 #14744;Matejčić, 2015 #14745;Oh, 2015 #14743;Potini, 2015 #14741;Sampath, 2015 #14746;Sun, 2015 #14747;Aksahin, 2016 #14732;Camarda, 2016 #14737;Hermansen, 2016 #14736;Kadar, 2016 #14740;Kataoka, 2016 #14735;Verbeek, 2016 #14738;Wild, 2016 #14739;Yoon, 2016 #14734;Zhang, 2016 #14733;Ali Yousef, 2017 #14723;Damrow, 2017 #14727;Fan, 2017 #14729;Massoud, 2017 #14728;Meng, 2017 #14722;Siljander, 2017 #14726;Swensen, 2017 #14725;Wight, 2017 #14731;Yang, 2017 #14724;Zhang, 2017 #14730;Bradko, 2018 #14718;Cho, 2018 #14717;Cusano, 2018 #14720;Gu, 2018 #14713;He, 2018 #14715;Meng, 2018 #14716;Paxinos, 2018 #14714;Siljander, 2018 #14721;Zarins, 2018 #14719;Chen, 2019 #14712;Dissaneewate, 2019 #14709;Jang, 2019 #14707;Li, 2019 #14708;Schütte, 2019 #14711;Shea, 2019 #14710;Achudan, 2020 #14690;Fan, 2020 #14700;Gammon, 2020 #14705;Güler, 2020 #14703;Hijazin, 2020 #14694;Hsieh, 2020 #14698;Khidzhazin, 2020 #14704;Liu, 2020 #14697;Mehta, 2020 #14699;Misir, 2020 #14695;Schmidt-Hebbel, 2020 #14696;Siddiqui, 2020 #14693;Sun, 2020 #14692;Wilding, 2020 #14691;Wong, 2020 #14689;Yu, 2020 #14706;Zhang, 2020 #14702;Zhu, 2020 #14701;Bae, 2021 #14686;Bulaïd, 2021 #14688;Chang, 2021 #14664;Deng, 2021 #14663;Fehske, 2021 #14673;Harna, 2021 #14674;He, 2021 #14679;Jang, 2021 #14676;Jang, 2021 #14677;Kfuri, 2021 #14681;Kim, 2021 #14669;Lu, 2021 #14667;Meng, 2021 #14678;Mengana, 2021 #14672;Ngissah, 2021 #14680;O’Donnell, 2021 #14687;Raoulis, 2021 #14665;Shi, 2021 #14671;Sidharthan, 2021 #14683;Sim, 2021 #14675;Sousa, 2021 #14684;Tanpowpong, 2021 #14666;Wang, 2021 #14670;Xie, 2021 #14682;Yu, 2021 #14668;Yu, 2021 #14685;Berninger, 2022 #14662;Chao, 2022 #14658;Chen, 2022 #14642;Devana, 2022 #14656;Du, 2022 #14647;Gao, 2022 #14651;Gao, 2022 #14645;Gu, 2022 #14644;Hannah, 2022 #14654;Hu, 2022 #14655;Kuo, 2022 #14640;Li, 2022 #14641;Li, 2022 #14649;Lin, 2022 #14659;Lowe, 2022 #14650;Negrão, 2022 #14660;Park, 2022 #14638;Pu, 2022 #14639;Raja, 2022 #14643;Seggewiss, 2022 #14637;Tengler, 2022 #14648;Xie, 2022 #14657;Zhang, 2022 #14653;Zhou, 2022 #14661;Zhu, 2022 #14652;Zhu, 2022 #14646;Englert, 2023 #14633;Jian, 2023 #14630;Liu, 2023 #14631;Liu, 2023 #14632;Ma, 2023 #14635;Mei, 2023 #14634;Vesseur, 2023 #14629;Yan, 2023 #14636} |
