# Supplementary material for: Surgical treatment of inferior pole fractures of the patella: a systematic review
Source: J Exp Orthop. 2023 Jun 1;10:58. doi: 10.1186/s40634-023-00622-y (PMC10234962; doi:10.1186/s40634-023-00622-y)
Supplement: Supplementary file 2 — Additional file 2: Appendix 2. Detail of the articles. [file 40634_2023_622_MOESM2_ESM.docx]

Appendix 2 Detail of the article

| Author (Year) | Method | No. of patients | Start time of rehabilitation (weeks) | | Surgical time (mins) | Radiologic outcomes | | Functional outcomes | | | Complications | ROIs |
| --- | --- | --- | --- | --- | --- | --- | --- | --- | --- | --- | --- | --- |
|  |  |  | ROM | Weight bearing |  | Union time (weeks) | Patella height measurement | ROM (degree) | Functional score | |  |  |
| **Rigid fixator** | | | | | | | | | | | | |
| Kastelec M (2004) | Plate | 11 | 0 | 0 | NM | NM | BP index | NM | Patello-femoral Rating System | 94.1 ± 5.9 | 0 | 7 |
| Matejcic A (2006) | Plate | 51 | NM | NM | NM | NM | NM | NM | Modifying Cinncinati knee rating system | 91 | 0 | 51 |
| Matejcic A (2008) | Plate | 71 | 0 | 0 | NM | NM | NM | NM | Modifying Cinncinati knee rating system | NM | NM | 71 |
| Liu XW (2011) | Concentrator | 25 | 0 | 0 | 57.8 ± 8.7 | 6.1 ± 0.9 | NM | NM | Bostman score | 29.5 ± 0.9 | 1 | 25 |
| Matejcic A (2015) | Plate | 98 | 0 | 0 | 52.5 | NM | NM | NM | Modifying Cinncinati knee rating system | 89 | 3 | 98 |
| Du B (2022) | Plate + cerclage wire | 28 | 0 | NM | NM | NM | NM | 126.8 ± 4.9 | Bostman score | 28.6 ± 1.1 | 0 | 1 |
| Gu H (2022) | Plate + cerclage wire | 16 | 0 | 0 | 62.2 ± 13.3 | 10.8 ± 2.4 | NM | 127.5 ± 13.9 | Bostman score | 27.8 ± 3.0 | 1 | 11 |
| Li M (2022) | Plate + cerclage wire | 21 | NM | NM | 75.1 ± 7.3 | 9.3 ± 1.6 | NM | 131.4 ± 6.9 | Bostman score | 28.7 ± 1.2 | 0 | NM |
|  |  |  |  |  |  |  |  |  | Lysholm score | 89.2 ± 4.5 |  |  |
| Chen R (2022) | Concentrator | 46 | 0 | NM | NM | NM | IS index | NM | Bostman score | NM | 1 | NM |
| Chen R (2022) | Concentrator + cerclage wire | 48 | 0 | NM | NM | NM | IS index | NM | Bostman score | NM | 0 | NM |
| Ma XY (2023) | Plate | 30 | 0 | 2 | 76.2 ± 15.3 | 6.1 ± 0.9 | NM | 123.3 ± 9.8 | Bostman score | 26.8 ± 2.1 | 1 | 0 |
| **Tensile device dominant: SVM** | | | | | | | | | | | | |
| Yang KH (2003) | SVM | 25 | 0 | 0 | 82.0 ± 17.4 | 7.1 ± 1.3 | NM | NM | Bostman score | 29.5 ± 0.9 | 1 | 6 |
| Kim YM (2011) | SVM | 18 | 4 | 0 | NM | 13.8 ± 2.1 | NM | 136.3 ± 3.0 | Bostman score | 28.6 ± 2.3 | 0 | NM |
| Song HK (2014) | SVM + cerclage wire | 21 | 0 | 0 | NM | 7.9 ± 1.3 | Plateau-patella angle | 138.3 ± 5.8 | Bostman score | 28.1 ± 1.2 | 0 | 7 |
| Oh HK (2015) | SVW + TOR | 11 | 1 | 0 | NM | NM | NM | 129.4 ± 6.3 | Bostman score | 29.6 ± 0.6 | 0 | 4 |
| Fan J (2017) | Modified SVW | 11 | 0 | 2 | NM | 12 | Patella height | 130.2 ± 5.8 | Bostman score | 28.2 ± 0.8 | 0 | NM |
| Cho JW (2018) | SVW + Plate buttress | 13 | 0 | 2 | 71.1 ± 5.3 | 10.2 ± 2.0 | NM | 127.7 ± 4.2 | Bostman score | 29.6 ± 0.9 | 1 | NM |
| He S (2018) | SVW + cerclage wire | 11 | 0 | 0 | 44.4 ± 13.0 | NM | NM | 130.9 ± 2.9 | Bostman score | 29.1 ± 0.8 | 0 | NM |
| Yu H (2021) | SVW + cerclage wire | 23 | 0 | NM | 76.3 ± 7.9 | <10 | NM | 125.7 ± 6.9 | Bostman score | 28.1 ± 1.2 | 0 | 2 |
| Yan SG (2021) | SVW + cerclage wire | 20 | 0 | 0 | 45.7 ± 8.8 | NM | NM | 131.3 ± 3.5 | Bostman score | 29.4 ± 0.7 | 0 | 20 |
| **Tensile device dominant: TOR** | | | | | | | | | | | | |
| Kastelec M (2004) | TOR with PP | 13 | 7 | 0 | NM | NM | NM | NM | Patello-femoral Rating System | 81.2 | 1 | 0 |
| Matejcic A (2008) | TOR with PP | 49 | 5 | 0 | NM | NM | NM | M, | Modifying Cinncinati knee rating system | NM | NM | 0 |
| Kadar A (2016) | TOR | 33 | 6 | 0 | 79.1 ± 17.8 | NM | NM | 107.3 ± 7.22 | SF-12 PCS | 40.3 ± 12.5 | 3 | NM |
|  |  |  |  |  |  |  |  |  | SF-12 MCS | 50.3 ± 13.1 |  |  |
|  |  |  |  |  |  |  |  |  | Kujala score | 69.0 ± 24.6 |  |  |
| Massoud EIE (2017) | TOR + cerclage wire | 23 | 0 | 0 | NM | 9.8 ± 2.0 | BP index | NM | Bostman score | 28.1 ± 2.8 | 0 | 23 |
| Achudan S (2020) | TOR + figure 8 wire | 14 | 0 | 0 | NM | NM | NM | 115.2 | NM |  | 0 | NM |
| Chang CH (2021) | TOR | 25 | 2 | 0 | NM | NM | IS index | NM | NM |  | 3 | 0 |
| Huang WZ (2021) | TOR | 14 | 4 | 2 | 62.3 ± 6.4 | NM | NM | 133.6 ± 6.3 | Bostman score | 28.7 ± 1.3 | 0 | NM |
|  |  |  |  |  |  |  |  |  | Lysholm score | 94.4 ± 3.9 |  |  |
| Jang JH (2021) | TOR+ plate buttress | 12 | 4 | NM | NM | 8.6 ± 1.6 | Patella height | 129.6 ± 5.2 | NM |  | 0 | NM |
| Hu JL (2022) | TOR + cerclage wire | 22 | NM | NM | NM | NM | IS index | 138.2 ± 5.9 | Lysholm score | 95.9 ± 3.6 | 0 | NM |
| Kuo LY (2022) | TOR | 15 | 0 | 0 | NM | NM | CD index | 110.0 ± 18.9 | NM |  | 0 | 0 |
| Zhou M (2022) | TOR + suture bridge | 18 | 1 | 6 | NM | 10.1 ± 2.2 | IS index | 135.8 ± 8.8 | Bostman score | 28.9 ± 1.1 | 0 | NM |
| **Tensile device dominant: SA** | | | | | | | | | | | | |
| Kadar A (2016) | SA | 27 | 6 | 0 | 68.5 ± 19.8 | NM | NM | 112.5 ± 7.5 | SF-12 PCS | 45.1 ± 11.9 | 4 | NM |
|  |  |  |  |  |  |  |  |  | SF-12 MCS | 53.3 ± 8.0 |  |  |
|  |  |  |  |  |  |  |  |  | Kujala | 74.3 ± 26.2 |  |  |
| Huang WZ (2021) | SA | 21 | 4 | 2 | 41.1 ± 4.2 | Almost 12 | NM | 129.8 ± 6.2 | Bostman score | 27.8 ± 1.4 | 0 | NM |
|  |  |  |  |  |  |  |  |  | Lysholm score | 91.4 ± 5.1 |  |  |
| Kim KS (2021) | SA | 22 | 4 | NM | NM | 19 | IS index | 130.0 ± 7.0 | IKDC | 71 | 1 | 2 |
|  |  |  |  |  |  |  |  |  | KOOS | 82 |  |  |
|  |  |  |  |  |  |  |  |  | Lysholm | 85 |  |  |
| Yu H (2021) | SA | 25 | 0 | NM | 46.5 ± 6.43 | NM | IS index | 124.8 ±7.2 | Bostman score | 28.3 ± 1.3 | 0 | 0 |
| Xie J (2022) | SA + figure 8 wire | 10 | 0 | 0 | NM | NM | NM | 112.0 ± 17.8 | Bostman score | 28.3 ± 2.2 | 1 | NM |
| Park YG (2022) | SA | 28 | 4 | 0 | NM | 15.2 ± 4.6 | IS index | 129.6 ± 5.8 | Lysholm score | 94.4 ± 3.4 | 0 | 0 |
|  |  |  |  |  |  |  |  |  | Kujala | 88.9 ± 3.4 |  |  |
| **Mixed device: TBW** | | | | | | | | | | | | |
| Chang SM (2011) | Screw + TBW | 10 | 0 | 0 | NM | NM | NM | 123.0 ± 13.6 | Bostman score | 28.7 ± 1.3 | 0 | 6 |
| Yang X (2017) | TBW + cerclage wire | 11 | 0 | 0 | NM | NM | NM | 129.6 ± 3.5 | Rasmussen scores | 27.9 ± 0.6 | 0 | 8 |
| Li J (2019) | TBW | 28 | NM | NM | 88.3 ± 11.8 | 12.1 ± 1.9 | NM | 98.21 ± 21.7 | NM |  | 0 | NM |
| Zhu W (2020) | TBW + plate buttress | 17 | 0 | 2 | NM | 9.7 ± 1.7 | NM | 128.2 ± 8.6 | Bostman score | 27.5 ± 2.5 | 0 | 5 |
| Zhang ZS (2020) | Screw TBW | 41 | 0 | 0 | 76.4 ± 18.0 | 9.6 ±1.9 | NM | 126.7 ± 6.6 | Bostman score | 28.5 ± 1.6 | 0 | NM |
| Chang CH (2021) | TBW | 30 | 2 | 0 | NM | NM | IS index | NM | NM |  | 2 | 18 |
| Lu MK (2021) | TBW + SA | 17 | 0 | 0 | 82.4 ± 9.7 | NM | NM | 129.4 ± 8.6 | Modified Cincinnati | 90.4 ± 4.4 | 0 | 2 |
| Lu MK (2021) | TBW | 20 | 0 | 0 | 54.4 ± 10.5 | NM | NM | 123.5 ± 13.2 | Modified Cincinnati | 87.9 ± 8.9 | 3 | NM |
| Du B (2022) | TBW | 21 | NM | NM | NM | NM | NM | 116.5 ± 8.7 | Bostman score | 24.8 ± 1.1 | 1 | 3 |
| Xie J (2022) | TBW | 18 | 0 | 0 | NM | NM | NM | 96.7 ± 11.8 | Bostman score | 24.7 ± 2.0 | 3 | NM |
| Gao Z (2022) | TBW + TOR | 15 | NM | NM | NM | NM | NM | 128.5 ± 7.1 | Bostman score | 28.4 ± 1.3 | NM | 9 |
| Jian Z (2022) | TBW + cerclage wire | 31 | 0 | 2 | 50 | 8 | NM | 120 (115-130) | Bostman score | 29.0 (26.5-30) | 0 | 0 |
| Park YG (2022) | TBW | 35 | 4 | 0 | NM | 14.6 ± 4.9 | IS index | 128.8 ± 8.3 | Lysholm score | 92.4 ± 5.4 | 0 | 35 |
|  |  |  |  |  |  |  |  |  | Kujala | 87.2 ± 5.8 |  |  |
| **Mixed device: Other** | | | | | | | | | | | | |
| Liu CD (2023) | Screw + SA | 22 | 0 | 1 | 78.8 ± 11.2 | 11.2 ± 1.6 | NM | 136.7 ± 4.7 | KOOS | 86.00 ± 5.2 | 0 | NM |
|  |  |  |  |  |  |  |  |  | Bostman score | 28.3 ±1.4 |  |  |
| **Extra-patella device: PT wire** | | | | | | | | | | | | |
| Singh RP (2007) | TOR with PP + PT | 14 | 0 | 0 | NM | NM | BP index | NM | Patellofemoral scoring system | 94.1 | NM | 14 |
| Li J (2019) | TBW + cerclage wire + PT | 30 | NM | NM | 92.1 ± 1.4 | 12.5 ± 1.4 | NM | 111.3 ± 13.0 | Bostman score | NM | 0 | NM |
| Zhang ZS (2020) | Screw TBW + PT | 22 | 3 | 0 | 64.2 ± 10.8 | 11.4 ± 1.3 | NM | 117.3 ± 6.9 | Bostman score | 25.8 ± 1.3 | 0 | NM |
| Kuo LY (2022) | TOR + PT | 20 | 0 | 0 | NM | NM | NM | 108.0 ± 23.8 | NM |  | 2 | 4 |
| **Extra-patella device: ESF** | | | | | | | | | | | | |
| Pu SQ (2022) | Suture + ESF | 11 | 0 | 0 | 56.4 ± 8.4 | 8.9 ± 1.5 | NM | 129.7 ± 3.3 | Bostman score | 29.2 ± 1.0 | 0 | NM |

BP index, Blackburne–Peel index; CD index, Caton–Deschamps index; ESF, external skeletal fixator; IS index, Insall–Salvati index; KOOS, Knee Injury and Osteoarthritis Outcome Score; NM, not mentioned; PP, partial patellectomy; PT, patellotibial wire; SA, suture anchor; SVW, separated vertical wire; TBW, tension band wire; TOR, transosseous reattachment
